# Supplementary material for: Simulation-based inference for non-parametric statistical comparison of biomolecule dynamics
Source: PLoS Comput Biol. 2023 Feb 2;19(2):e1010088. doi: 10.1371/journal.pcbi.1010088 (PMC9928078; doi:10.1371/journal.pcbi.1010088)
Supplement: S1 Text — Details about the MDS with uncertainty, the GNN architecture and training, the comparison with other statistical tests, as well as a study of the influence of the training dataset and supervised task. (PDF) [file pcbi.1010088.s001.pdf]

S1 Text

Multi-dimensional scaling with uncertainty on estimated distances

When estimating $\text{MMD}_u^2[\mathcal{F}, p, q]$ from sets of trajectories $X \sim p$ and $Y \sim q$, the uncertainty directly depends on the number of samples in both X and Y. In our case, the uncertainty, which can be evaluated by bootstrapping, is sometimes of the same order of magnitude than the estimated value. Furthermore, the number of elements per set (here, the number of trajectories per synapse), spans more than an order of magnitude and uncertainty thus greatly varies from one measure to the other. This should be taken into account when using $\text{MMD}_u^2[\mathcal{F}, X, Y]$ to embed subsets of trajectories.

Hence, starting from a matrix of squared distances $\mathbf{D}^2$ between N sets of trajectories, and a matrix of uncertainties of these squared distances $\mathbf{D}_\sigma^2$, we obtain a set of N Euclidian vectors $\{\mathbf{x}_1, \ldots, \mathbf{x}_N\}$ by maximizing the probability of the resulting squared distances, assuming that they follow Gaussian laws whose means are the coefficients of $\mathbf{D}^2$ and standard deviations coefficients of $\mathbf{D}_\sigma^2$. This amounts to solving the following optimisation problem :

$$\max_{\mathbf{x}_1,\ldots,\mathbf{x}_N} \sum_{i<j} \log\left(P_{i,j}\left(||\mathbf{x}_i - \mathbf{x}_j||^2\right)\right)$$

$$\max_{\mathbf{x}_1,\ldots,\mathbf{x}_N} \sum_{i<j} \left(\frac{||\mathbf{x}_i - \mathbf{x}_j||^2 - \mathbf{D}_{i,j}^2}{\mathbf{D}_{\sigma i,j}^2}\right)^2 .$$

We do so using a gradient ascent method.

Graph neural network features and architecture

Node and edge features

The features associated to nodes and edges of a trajectory's graph in the GRATIN summary network are defined using the following variables defined for each node $i \in \{1, \ldots, N\}$: the sum $R_i^{(k)}$ of step sizes elevated to the power k, up to point i: $\sum_{j\leq i} \|\Delta \mathbf{r}_j^k\|_2$. We denote as well s the standard deviation of step sizes. Then, the node features associated to a node i are:

1. the normalised time: i/N,
2. the normalised distance to origin $\|\mathbf{r}_i\|_2/s\sqrt{i}$,
3. the normalised maximal distance to origin up to point i : $\max_{k\leq i}\|\mathbf{r}_k\|_2/s\sqrt{i}$,
4. normalised cumulative powered distances covered by the walker up to point i, with $k \in \{1, 2, 4\}$: $\frac{N}{i}\frac{R_i^{(k)}}{R_N^{(k)}}$.

The six features associated to an edge (i, j) with $i < j$ are:

1. the time difference: $j - i$,
2. the normalised distance between edge source and target: $\|\mathbf{r}_j - \mathbf{r}_i\|_2/s\sqrt{j-i}$,
3. the normalised dot product of jumps: $\Delta \mathbf{r}_i.\Delta \mathbf{r}_j/s^2$,

4. normalised differences of the cumulative powered distances covered by the walker from point i to j, with $k \in \{1, 2, 4\}$: $\frac{N}{j-i}\frac{R_j^{(k)} - R_i^{(k)}}{R_N^{(k)}}$.

The number of operations required to compute features scales linearly with the trajectory length. A trajectory-level feature accounts for the trajectory scale: the standard deviation of step sizes. It is appended at the end of t

GNN architecture

The architecture of the GNN used in the summary network is similar to the encoder network proposed in [1] and the same as in [2], with the difference that we here additionally apply edge features. Node and edge features are first passed to multi-layer perceptrons, which embed them in an 8-dimensional space. The network is then composed of three successive convolution layers, one relying simply on node features and the two others being conditioned by edge features. We used as convolutions the GIN layers introduced in [3]). They output $\mathbf{x}^{(1)}$, $\mathbf{x}^{(2)}$ and $\mathbf{x}^{(3)}$ vectors, each of 32 dimensions (equivalent to 32 convolution filters), which are concatenated to form $\mathbf{x}^{(f)}$. The rows of this $(N, 32 \times 3)$ matrix of nodes features are then aggregated using an attention mechanism during the pooling step, to keep just one row per graph, i.e., per trajectory. This vector is subsequently passed to a three-layer perceptron, the output of which is the 16-dimensional latent vector. The dimension of the latent space is voluntarily higher than that of the parameters space so as to facilitate the neural network convergence, which is helped by over-parameterisation [4]. All multi-layer perceptrons have a leaky-ReLU activation with slope 0.1 for negative values. We summarise their shapes in S1 table, for a total of 52 590 network parameters.

Comparison with other statistical tests

We performed a series of tests to compare the efficiency of :

- the MMD test compared to $t-$test (1D vectors) or Hotelling test ($D \geq 2$)
- various types of descriptive statistics (based on analytically defined quantities, outputs of neural networks, or latent variables) Results are shown in the figure below
  - $\log_{10}(D)$: log of the diffusivity estimated using the formula presented in [5]
  - Alpha: anomalous diffusion exponent estimated using our neural network
  - Convex-hull: 3D vector composed of the trajectory's convex hull area, perimeter and diameter.
  - Fractal dimension: fractal dimension of the trajectory, as computed in [6]
  - Asymmetry, as defined in [6]

We performed our tests on three random walk models: sBM, fBM and CTRW. For each pair of models, we considered sets of trajectories composed of mixtures of the two, with proportions 25%/75% and 75%/25%. Trajectories were either "pure" (standard realisations of a model) or "intermittent" (i.e. switching once from one model to the other, so that the overall fraction of time spent in each model corresponds to the desired fraction for the set). Trajectories were simulated with $D = 10^{-1.5}$ um^2/s, length=15, exposure = 15ms and localization noise between 15 and 50nm. Sets were composed of 200 trajectories in total. For each pair of model, we had 4 sets (2 proportions of mixture $\times$ 2 ways of mixing trajectories). We performed comparisons between these sets and

recorded the p-values yielded by the different tests. We generated 10 versions of each set to average over repetitions.

The first conclusion of this experiment, shown in S4 Fig A, is that the MMD test yields on average lower $p-$values than the Hotelling/$t-$test, regardless of the type of features used or the comparison. This confirms what was observed by the authors of the MMD method in their introductory paper [7], where they show that the MMD-based bootstrap test is, in most cases, the best performing one among a set of pre-existing multivariate statistical tests. To ensure that neither of our tests were under-estimating p-values, we recorded the p-values obtained with each test when comparing two sets of trajectories obtained with the same process (25% sBM, 75% fBM), over 500 repetitions (re-generating trajectories each time). The cumulative probability distribution of p is shown in panel S4 Fig C, and corresponds in both cases to what it should be, given that H_0 is true.

In S4 Fig B, we show the performance of MMD tests based on the various types of descriptive features that we considered. Results of our experiments are displayed on the figure below. When a cell is red (resp. blue), it means "the vector of the row yielded on average lower (resp. larger) p-values than the vector of the column". For instance, the red top cell means "MMD tests based on alpha yielded lower p-values than those based on log(D)". It is clear from these experiments that vectors output by the neural networks carry more information than the analytical ones ($\log_{10}(D)$, fractal dimension, convex hull, asymmetry).

Overall, the best descriptive vector is the "Combined metrics" one, which is a mix of neural network outputs and analytical indicators and have a slight advantage over the Gratin 2D and 16D vectors. More compressed than the 16D Gratin or the 12D "combined metrics" vector, the Gratin 2D vector offers a good compromise between interpretability and richness of description.

Influence of the training method and data

We investigated the influence on the learnt 2D representation of trajectories of i) the choice of tasks used to train the model and ii) the choice of random walk types included in the training data. We show the results on S5 Fig We observe on S5 Fig A that the four types of random walks we consider are well separated even by a network trained on a single type of random walk (and better separated if we include more models). Besides, we see on S5 Fig B that when we only train the model to infer the anomalous exponent, the resulting latent space has a rather spindly shape. Even in this case, the various types of RW are well separated.

This suggests that simulation-based inference produces well-structured latent representations in many configurations, and hence that the results we obtained and presented in the present work are not critically dependent of the exact method of training we used.

References

1. Verdier H, Duval M, Laurent F, Cassé A, Vestergaard CL, Masson JB. Learning physical properties of anomalous random walks using graph neural networks. Journal of Physics A: Mathematical and Theoretical. 2021;54(23):234001.

2. Verdier H, Laurent F, Cassé A, Vestergaard CL, Masson JB. Variational inference of fractional Brownian motion with linear computational complexity. Physical Review E. 2022;106(5):055311.

3. Xu K, Hu W, Leskovec J, Jegelka S. How powerful are graph neural networks? arXiv preprint arXiv:181000826. 2018;.

4. Du SS, Zhai X, Poczos B, Singh A. Gradient descent provably optimizes over-parameterized neural networks. arXiv preprint arXiv:181002054. 2018;.

5. Vestergaard CL, Blainey PC, Flyvbjerg H. Optimal Estimation of Diffusion Coefficients from Single-Particle Trajectories;89(2):022726. doi:10.1103/PhysRevE.89.022726.

6. Kowalek P, Loch-Olszewska H, Szwabiński J. Classification of diffusion modes in single-particle tracking data: Feature-based versus deep-learning approach;100(3):032410. doi:10.1103/PhysRevE.100.032410.

7. Gretton A, Borgwardt KM, Rasch MJ, Schölkopf B, Smola A. A kernel two-sample test. The Journal of Machine Learning Research. 2012;13(1):723–773.
