# Supplementary material for: Simulation-based inference for non-parametric statistical comparison of biomolecule dynamics
Source: PLoS Comput Biol. 2023 Feb 2;19(2):e1010088. doi: 10.1371/journal.pcbi.1010088 (PMC9928078; doi:10.1371/journal.pcbi.1010088)
Supplement: S1 Table — Sizes of multi-layer perceptrons used in the neural network. (PDF) [file pcbi.1010088.s002.pdf]

MLP	Layers
edge features embedding	(6,32,32,8)
node features embedding	(6,32,32,8)
convolution layers (x3)	(8,32,32, 32)
pooling layer	(96,32,1)
final embedding	(97, 32, 32, 48, 32)
alpha predictor	(16,64,64,1)
model classifier	(16,64,64,5)

S1 Table. Shapes of multi-layer perceptrons used in the network.
