# Supplementary material for: Simulation-based inference for non-parametric statistical comparison of biomolecule dynamics
Source: PLoS Comput Biol. 2023 Feb 2;19(2):e1010088. doi: 10.1371/journal.pcbi.1010088 (PMC9928078; doi:10.1371/journal.pcbi.1010088)
Supplement: Attachment — Submitted filename: Reviewers alphaSyn.pdf [file pcbi.1010088.s008.pdf]

PLoS Comp Biol submission no. PCOMPBIOL-D-22-00557

To whom it may concern,

We are happy for the opportunity to resubmit our manuscript to PLOS Computational Biology. The referees found the paper interesting and technically sound. However, they both had concerns about a lack of discussion of and comparison to existing state of the art. While we purposefully left out an extended comparison due to the conceptual and practical difference between our proposed methodology and existing ones, we added background elements allowing readers from other fields to understand the purpose of the method and the use case it addresses. We have consequently added an extensive discussion of how our methodology relates to existing ones as well as a quantitative comparison of its performance to tests based on descriptive statistics. Since the main focus of the article is the method, and not biological results, we have changed the title to "Simulation-based inference for non-parametric statistical comparison of biomolecule dynamics" to change the emphasis.

We believe that the present manuscript now provides a clearer motivation for our proposed framework as an approach that mixes the best parts of the two existing paradigms, model-based inferential approaches and tests based on descriptive statistics, as it combines the statistical power of the first with the absence of strong modelling assumptions of the second.

PLoS Comp Biol submission no. PCOMPBIOL-D-22-00557

Responses to reviewers

Reviewer #1:

Verdier et al describe a methodology for comparing sets of biomolecule trajectories as obtained in imaging experiments to identify changes in the associated dynamics. They propose a two-step scheme, involving feature extraction through machine learning (via a graph neural network) and a classical statistics test (maximum mean discrepancy). They apply this approach to detect differences in the dynamics of a presynaptic protein.

The task of extracting information from short and noisy trajectory is relevant and has been included in a recent competition in which the authors took part but they do not mention/cite (Muñoz-Gil et at, Nat Comms 2021). Unsupervised methods have also been recently proposed for this task (Muñoz-Gil JPhysA 54 504001 2021).

Not citing the ANDI challenge paper was an omission on our part. We now cite it in the revised manuscript. We also added a reference to the method of Muñoz-Gil et al. 2021, which, like ours, relies on learnt representations of individual trajectories.

The method they present in the manuscript builds on the one they developed for the competition and is based on graph neural networks (Verdier et al, JPhysA – ref 24). Recently, graph neural networks are getting a lot of attention and might potentially be a good strategy for this kind of problem. The results presented in the manuscript seem promising but are mostly presented at a qualitative level and lack comparison with a gold standard. It is difficult to understand the performance of the methods since very little quantitative assessment is presented.

I have a few major concerns:

1. Features are extracted at the single-trajectory level, but the comparison is done between sets of (short) trajectories. Is it really necessary to use such a complex architecture for this task? Wouldn't other classical approaches at the ensemble level perform similarly (i.e., comparing the distribution of D, or of displacements, or of gyration radii)? For example, Fig 6 suggests that differences are mainly due to a change in mobility (as also stated in the intro) that could be detected also with other methods.

The referee is correct that one may very well test for differences in the values of classic, handcrafted, features. However, our proposed approach enjoys two main advantages over such methods. First, the latent representation learned by the neural network is optimised to be maximally informative about random walk types and parameter values; a vector of handcrafted features is not optimised for statistical efficiency. Second, the MMD test leverages the continuous latent representation to provide a powerful test that does not rely on parametric assumptions like classical tests (e.g., t-test or Hotelling's t^2) do.

Based on the reviewer's interrogations, we performed a series of tests to compare the efficiency of :

- The MMD test VS t-test (1D vectors) / Hotelling (D > 1)

- Various types of descriptive statistics (based on analytically defined quantities, outputs of neural networks, or latent variables)

Results are shown in the figure below (Fig. S4 in the revised manuscript)

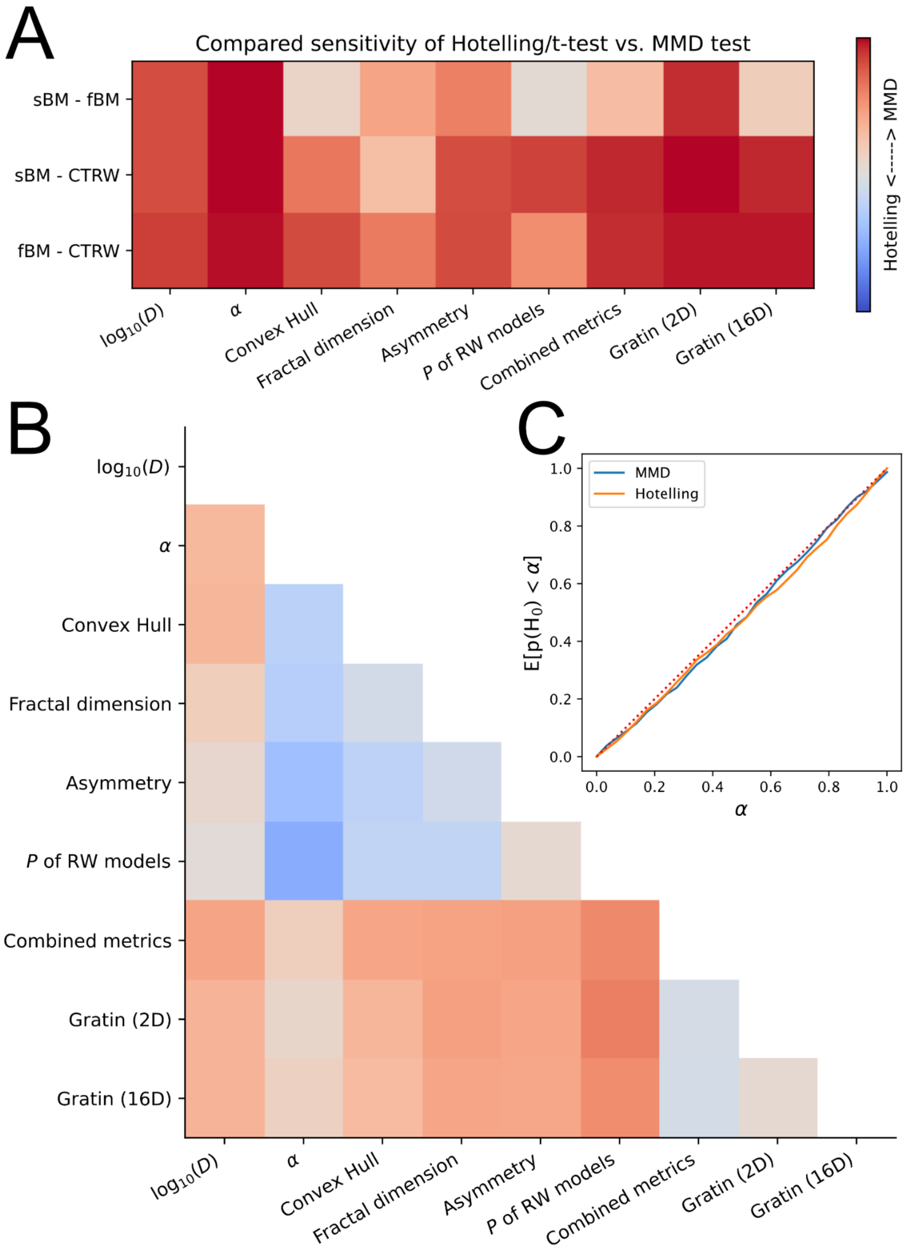

In panel A, cells are blue (resp. red) when the Hotelling test yields lower (resp. higher) p-values than the MMD test. Rows indicate the two types of random walks which are compared, columns correspond to various types of descriptive vectors used:

- log_10(D): log of the diffusivity estimated using the formula presented in Vestergaard 2014
- Alpha: anomalous diffusion exponent estimated using our neural network

- Convex-hull: 3D vector composed of the trajectory's convex hull area, perimeter and diameter.
- Fractal dimension: fractal dimension of the trajectory
- Asymmetry: asymmetry
- P of RW models: 5D vector composed of the probabilities (estimated using our neural network) of being a realisation of each model
- Combined metrics: 12D vector, concatenation of all of the above
- Gratin (2D): UMAP-compressed version of the latent vector learnt by our network
- Gratin (16D): latent vector learnt by the network.

For each pair of RW models, we considered sets of trajectories composed of mixtures of the two models, with proportions 0.25/0.75 and 0.75/0.25. Trajectories were either "pure" (standard realisations of a model) or "intermittent" (i.e. switching once from one model to the other, so that the overall fraction of time spent in each model corresponds to the desired fraction for the set). Trajectories were simulated with D = 10^{-1.5} um^2/s, length=15, exposure = 15ms and localization noise between 15 and 50nm. Sets were composed of 200 trajectories in total. For each pair of model, we had 4 sets (2 proportions of mixture * 2 ways of mixing trajectories). We performed comparisons between these sets and recorded the p-values yielded by the different tests. We generated 10 versions of each set to average over repetitions.

The conclusion of this experiment is that the MMD test yields on average lower p-values than the Hotelling/t-test, regardless of the type of features used or the comparison. To ensure that neither tests were not under-estimating p-values, we recorded the p-values obtained with each test when comparing two sets of trajectories obtained with the same process (25% sBM, 75% fBM), over 500 repetitions (re-generating trajectories each time). The cumulative probability distribution of p is shown in panel C, and corresponds in both cases to what it should be given that H_0 is true.

In panel B, we show the performance of MMD permutation tests based on the various types of descriptive features that we considered. Results of our experiments are displayed on the figure below. When a cell is red (resp. blue), it means "the vector of the row yielded on average lower (resp. larger) p-values than the vector of the column". For instance, the red top cell means "MMD tests based on alpha yielded lower p-values than those based on log(D)".

It is clear from these experiments that vectors output by the neural networks carry more information than the analytical ones (log_10(D), fractal dimension, convex hull, asymmetry). Overall, the best descriptive vector is the "Combined metrics" one, which is a mix of neural network outputs and analytical indicators. The Gratin 2D and 16D nonetheless perform good as well, and have the advantage over others (except for the 1D alpha vector) of being "homogeneous". More compact than the 16D Gratin or the 12D "combined metrics" vector, the Gratin 2D vector offers a good compromise between interpretability and richness of description.

Finally, wanting to focus on the generality and power of our approach, we have shifted the focus of the manuscript towards the method and emphasised less on its biological validation. The biological results are indeed not very surprising and the application is mainly meant to validate the method on experimental data. We have changed the title of the paper to

“Simulation-based inference for non-parametric statistical comparison of biomolecule dynamics” to reflect this.

2. The manuscript lacks a baseline with a classical approach and a comparison with other methods designed to tackle similar problems (e.g., the winners of the competition mentioned above). For example, it would be important to understand what the advantage (if any) is of using a graph neural network over a CNN or RNN for this task. This point should be demonstrated by comparing the performance of different architectures on the same set of data.

As stated above, we now include a discussion of and a numerical comparison to approaches based on classical statistics. We do not expect that using a CNN or RNN encoder network would significantly alter the performance of the test. Indeed, the use of a GNN is not integral to our methodology and any sufficiently powerful supervised representation learning technique would do. Deep learning methods are particularly efficient for representation learning, and the ANDI challenge has in particular demonstrated their efficiency for inferring properties of random walks, with RNNs and GNNs being particularly useful because they can be applied directly to trajectories of varying lengths. We chose a GNN-based encoder since the latent description that it learns has interesting properties, including a sensible representation of trajectories generated by models that the GNN was not trained on [Verdier JPA 2021], and since its performance is close to optimal (in the particular case of fractional Brownian motion where optimality can be quantified) [https://arxiv.org/abs/2203.07961 (In press at PRE)] . In this second reference, we also compared the performance obtained by our GNN architecture with that obtained by an encoder based on 1D CNNs followed by LSTMs (similar to that used by the winners of the ANDI competition): the difference of performance between the two architectures (on the estimation of alpha) is negligible, especially on short trajectories. The advantage of the graph-based encoder is that it naturally enforces some of the problem’s symmetries (invariance by rotation) and explicitly encodes various scales of temporal correlations.

3. The manuscript lacks an ablation study that is necessary to conclude that the method is performing well due to its good design rather than to the potential artifacts.

In the new version of the manuscript, the main result of the paper really is the statistical test and its generality. We have added in figure S4 a comparison with other tests, in conditions less favourable (in terms of number of trajectories) than those of our biological example, showing that the description of trajectories by vectors of learnt features provide more sensitive statistical tests than more conventional descriptors. If one wishes to check that a statistical test based on the Gratin vectors is not an artefact of a given variable, it is always possible to “marginalise” the test, by equalising the distributions of the suspect variable prior to doing the bootstraps. We do this by default with the trajectory length (which we know has an influence on the latent vectors, as on other descriptive statistics).

Regarding our biological example, figure S3 does provide a verification that the detected difference is indeed evenly spread across all fields of view, and that it is not due to an artefact present in one replicate. We added at the end of the results section a remark about how such

tests could be used to probe the heterogeneity of experimental data sets with various levels of granularity.

4. The architecture requires a more detailed description. It is not clear which features are passed to each node/edge (Only coordinates? Are they normalized? Time? Do they use any embedding?) nor how the features are updated (message-passing?) and aggregated. Figure 2 seems to suggest that the graph is fully connected, but I could not find any mention of this in the manuscript. Is this the case or any sparsification is applied?

We have added details about the neural network architecture (in the main text, with more details in the supplementary information) and refer to our previous publication for detailed explanations about graph convolution operations.

Each node receives a maximum of k=10 connections. If the trajectory is shorter than k, then the graph is half-complete (each node receives connections from all its predecessors). Otherwise, nodes are drawn following a geometric-causal pattern (we have added details about this in the main text). This means that the number of connections is bounded from above by kN.

5. The link to the code directs to a private repo. The reviewers must be enabled to check the code to better understand the method.

GRATIN encoder : https://github.com/hippover/gratin

Palmari (image processing, from .tif files to trajectories) : https://github.com/hippover/palmari/

MMD comparisons : We made the code of the palm-tools repo accessible to the public : https://gitlab.pasteur.fr/hverdier/palm-tools

We also released a web platform (currently in beta) allowing researchers to perform the kind of analysis presented in this paper on their data: https://tracktor.pasteur.cloud/

Notebook where the analysis is performed, using the previously mentioned codes : https://gitlab.pasteur.fr/hverdier/palm-tools/-/blob/master/examples/MMD-paper.ipynb

6. Does feature extraction provides any interpretability besides a hint about the generative model? A representation of the UMAP features with respect to the anomalous diffusion exponent (instead of D) might be helpful in this sense.

We added in figure 3 a mapping of alpha, the random walk model, and the log-diffusion, both on simulated trajectories (true values) and on experimental ones (inferred values). Panel J gives an intuition of the layout of trajectories throughout the latent space.

We also added in figure 6 a series of histograms showing the distribution of several descriptive quantities for trajectories found in two distinct regions of the latent space.

7. Linked to the previous comment: The additional statistical test somewhat subdues the elegance of an end-to-end trainable model. Is it really necessary? In the end, the results are based on a “soft” classification. In an equivalent fashion, one might think to perform

a regression and/or a classification tout court and then just compare the proportion of trajectories in each class.

To investigate this, we showed in figure S4 that statistical tests based on the 2D gratin vector are as sensible than those based on raw predictions of the network (probabilities of belonging to each class of model, alpha).

Then, it is in general better to clearly separate the process through which statistical descriptors are obtained from the subsequent statistical test, hence the somewhat inelegant two-step method presented here (one could also have thought of a network directly optimised to maximise the MMD, but this would have raised other difficulties and induces Type II errors)

8. Can't similar results be directly obtained by extracting global features from the graph neural network?

We are not entirely sure what the referee means by global features here. If it refers to the output of the GNN, **X** and **Y**, then these cannot be used directly as their size is proportional to the trajectory length and thus differ between trajectories. Thus a fixed-size feature vector is needed. This could very well be a hand-selected set of global functions of the node and link feature vectors. However, this would be suboptimal. What the pooling layer does instead is combine node features into a fixed-size vector in a manner that is optimised to contain as much information about random walk type and parameters as possible.

9. Many references miss journal, page, and year. Relevant literature about graph neural networks is missing (e.g., arXiv:1806.01261)

The reviewer is right, we fixed those.

Reviewer #2: In their manuscript the authors introduce a two-step statistical testing scheme combining simulation-based inference to train a neural network and a maximum mean discrepancy statistical test on the vectors of learned features to compare the features. They characterize sets of simulated random walks and analyze experimental alpha-synuclein traces in synapses in cultured cortical neurons in response to membrane depolarization. The authors also identify the domains in the latent space where the differences between biological conditions are the most significant,

Technically the work seems to be interesting and sound. There are, however, serious conceptual flaws in the motivation, (alleged) state of the art, and even the basic logic of their reasoning (for details please see below).

Several crucial (and recurring) statements are very puzzling, e.g. "… to detect changes in biomolecule dynamics within organelles without needing to identify a model of their motion". I cannot identify any reason whatsoever why it should be required to identify a model of motion in order to detect differences. Yet, this seems to be the main motivation for the work.

Specific remarks:

1) Basically the authors argue that instead of "describing trajectories using a set of explicitly defined features" (i.e. physics based observables) it is preferable to use a black box. The entire reasoning seems to be based on the premise that one is required to identify some underlying model for the dynamics in order to be able to identify and quantify changes in the dynamics, which is certainly not true. To spell this out, there is in fact no need for an underlying model if one sets out to quantify the properties of trajectories or to detect and quantify differences between them. There is a very large (and I claim open) set of physical observables one may infer directly from trajectory data in a model free fashion. For example, the "canonical" time averaged squared displacement, position power spectra, van Hove functions and single-trajectory van Hove functionals, occupation measures, asphericity of individual trajectories, spectral and fractal dimensions of trajectories, memory kernels and other memory quantifiers, etc. None of these requires any kind of underlying model, all of these directly provide physical intuition, in contrast to the proposed new approach.

The motivation and declared superiority (if any at all) of the proposed black-box approach must be quantified by a fair benchmark comparison (which may be difficult), or the statements claiming superiority dropped. Moreover, any kind of such statements must also be formulated precisely and factually. No comparison analysis is performed whatsoever. It is not clear if the above intuitive "canonical" measures for quantifying the properties of trajectories (and their differences) are truly outperformed by the present approach to a degree that would outweigh the fact that the black-box treatment offers little if any physical insight.

There are indeed a variety of quantitative properties of random walks which are of great value when analysing very long trajectories or homogeneous sets of short ones. But observations of biomolecule trajectories such as those acquired via PALM do not meet these criteria (details below). We added explanations on this topic in the revised manuscript, as – the reviewer is right – such arguments are essential to motivate one to resort to a method like ours.

Obtaining precise estimates of the descriptive statistics mentioned by the reviewer requires averaging, either over ensemble of trajectories or over time windows of a same trajectory, if one is interested in trajectory-level information. Very often, observed biomolecules do not all exhibit the same type of dynamics (and one might thus not want to assume that they do prior to analyse them) : some might engage in interactions depending on their conformation or on local properties of the cytoplasm. This diversity is interesting and averaging over all trajectories would prevent one from studying it further. On the other hand, trajectories acquired using PALM (such as those of alpha-synuclein which we observe) have a typical length of 10-20 points, preventing one from obtaining good estimates of correlation kernels, trajectory functionals which are functions of space and time, and even mean square displacement beyond one or two time lags. Besides, some statistics only make sense under the hypothesis of stationarity, which does not necessarily hold for biomolecule dynamics (although it is not, formally speaking, an identification of the model, the assumption of stationarity is a strong one).

Nevertheless, we included some of the descriptive statistics suggested by the reviewer (and which we could compute at the single trajectory level) in the experiments we did to compare the sensitivity of statistical tests based on various types of descriptive vectors. Results suggest that tests are not more sensible when based on these quantitative traits.

Finally, we vigorously disagree with the referee's statement that the proposed method does not provide any physical intuition. As we showed in Verdier et al. J Phys A, 2021, a trajectory's projected position in the latent space is directly related to its physical properties (see in Fig. 10 herein). In particular, by projecting onto the positions of numerical data, we can identify the most closely corresponding random walk model or models and associated physical parameter values. We enriched figure 3 and 5 so as to emphasise on the physical interpretation of the latent space's layout.

2) The existing literature on quantifying properties of individual particle traces is literally not existent. On the one hand this is nominally unacceptable. On the other hand it may explain the authors' belief that the task requires to identify some underlying model in the sense that they are perhaps simply not familiar with the literature well enough to recognize that this is not true.

We fully agree with the referee that the previous version of our manuscript lacked a discussion of existing non-parametric descriptive statistics, many of which have a long history in the SPT field. As we now discuss in the revised version of our manuscript, the price one pays for resorting to our method is the clarity of the interpretation, because the variables do not explicitly correspond to physical quantities. This price however offers a more generic approach and a better sensitivity if one has no prior knowledge of the observed system, orienting the analysis towards one or the other descriptive quantity. We argue however that the latent space is well structured and that, although less directly than with conventional descriptive statistics, a physical interpretation of detected differences can be recovered (see panels G-K of Fig. 6).

3) Consider the following conclusions stated in the manuscript:

"This indicates that the addition of KCl to the medium can affect the physical properties of many if not all alpha-synuclein molecules in a similar manner, irrespective of their subcellular location."

"This demonstrates that alpha-synuclein is highly mobile in living cells …".

"… the representative αlpha-synuclein trajectories exhibit a greater mobility in the depolarised state. This is likely the result of a weaker binding of alpha-synuclein at synapses, as reflected in the overall reduction of alpha-synuclein molecules during KCl application ..."

Any of the above mentioned canonical analyses would have directly reveal this information. In other words, the authors seem to have found a (very indirect) black-box substitute to infer physics. I am no biologist but these conclusions also do not seem to provide any new biological insight.

We changed the title of the paper and its general message to insist more on the method than on its biological validation. The reviewer is right that the difference in diffusivity is of such magnitude relative to the data abundance that other simpler methods would have detected it.

Nonetheless, we showed on simulated trajectories (Fig S4, shown below) that the method outperforms more conventional tests when used in less favourable conditions (i.e. with fewer trajectories). The experiments are described in detail in supplementary material.

Panel A: red means that MMD permutation test has more power than Hotelling/t-test.

Panel B: red means that the set of descriptive vectors from the row performs better than the one from the colum.

Panel C: cumulative distribution of the p-value yielded by the two tests on 500 repetitions of comparison of two sets of trajectories generated using the same models.

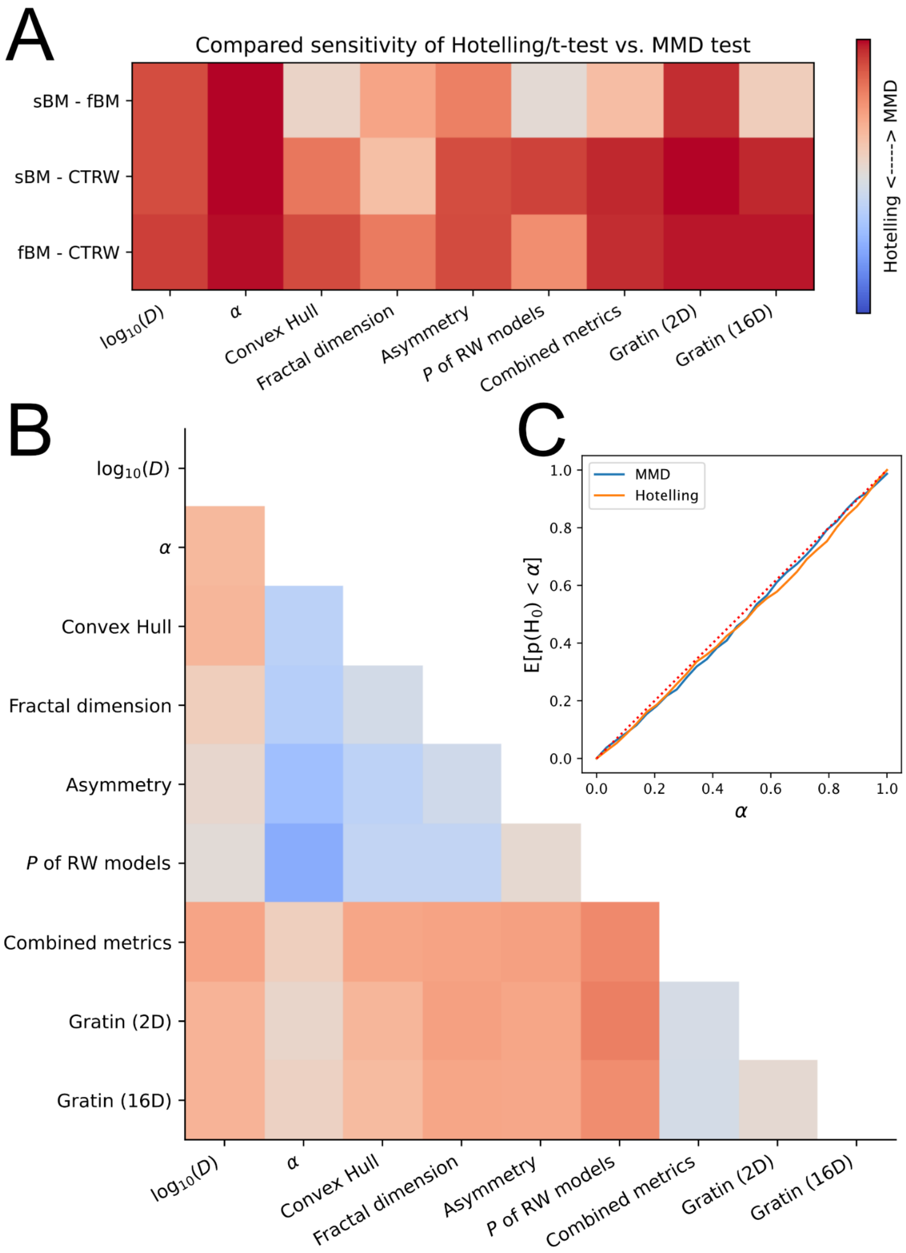

4) The title and discussion claims "subtle differences in alpha-synuclein dynamics". I am no biologist (perhaps this may be required) but I really struggled to identify why the observed differences are "subtle". Based on the title I expected that all canonically used methods employed in studies of particle transport (incl. the more "advanced" approaches) would fail to the detect differences, and this would call for the proposed approach. I would agree that such differences were subtle. But since no true comparison is presented the word "subtle" does not seem to be justified.

Similarly to the previous answer, the reviewer is right that the difference of diffusivity could have been detected by other means and we changed the title and message of the paper to insist more on the generality of the method and its potential to detect more subtle differences (with benchmarks on numerical data, see Fig. 4 and Fig. S4). The subtlety could also be justified by the fact that our methodology allows the comparison of sets composed of relatively few (100-200) trajectories, as we do in Fig. 7 when comparing synapses.

Summarizing remarks:

The results may certainly be interesting for a specialized community interested in technical aspects of the analysis of particle-tracking data. However, the analysis does not seem to be really required (at least with the motivation given in the manuscript). The way the results are presented one may be led to thinking that this is the kind of analysis to be performed if one aims to identify differences between measured trajectories but one does *not* want to gain the physical insight that comes "for free" in the application of traditional physics-based analyses. I may be mistaken, but the way the results are presented simply leads to such a conclusion.

As a result, based on this version of the manuscript I tend to recommend against publication and to instead seek a more specialized journal.

We disagree with the reviewer and added material in the paper to support our views. The latent space layout does rely on physical quantities, as shown in the panels we added in Fig. 6. The use case for which this analysis is intended is that of a biologist observing a biomolecule about which little is known, preventing him from making strong assumptions about the observed motion (such as stationarity, for TA-MSD analysis for instance, or homogeneity, on which ensemble averages rely).

Secondly, although here applied to the analysis of single particle tracking experiments, we think that the idea of using descriptive statistics obtained via simulation-based inference to perform statistical tests could well be adapted to other biological questions, and thus be of interest to non-specialists.

Have the authors made all data and (if applicable) computational code underlying the findings in their manuscript fully available?

The PLOS Data policy requires authors to make all data and code underlying the findings described in their manuscript fully available without restriction, with rare exception (please refer

to the Data Availability Statement in the manuscript PDF file). The data and code should be provided as part of the manuscript or its supporting information, or deposited to a public repository. For example, in addition to summary statistics, the data points behind means, medians and variance measures should be available. If there are restrictions on publicly sharing data or code —e.g. participant privacy or use of data from a third party—those must be specified.

Reviewer #1: **No:** The code and the data can't be accessed

Reviewer #2: Yes
