# Supplementary material for: Simulation-based inference for non-parametric statistical comparison of biomolecule dynamics
Source: PLoS Comput Biol. 2023 Feb 2;19(2):e1010088. doi: 10.1371/journal.pcbi.1010088 (PMC9928078; doi:10.1371/journal.pcbi.1010088)
Supplement: Attachment — Submitted filename: Reviewers alpha-syn (round 2)-3.pdf [file pcbi.1010088.s009.pdf]

# Reviewer #1

***(In black, your comments,** in purple, our answers**)***

**The revision has largely improved the manuscript but has produced a major shift in the focus of the article, which now reads as an example of a general recipe about how to handle relatively large datasets involving heterogeneous entities (in this case, sets of short single-molecule trajectories) by extracting meaningful features (using GNN, others ML approaches, or even analytical indicators), statistically testing them for (typically small) differences, and highlighting regions of the latent spaces that might produce these differences.**

**Due to the recent development of high-throughput methods for live-cell imaging and the inherent biological variability of these experiments, I believe this work is relevant to the quantitative development of the field.**

**Still, there are a few important points that, in might opinion, need to be further discussed and clarified. Since the authors are now focusing on the methodology and on its generality, I believe that it would be beneficial to provide additional insights on the statistical test and the simulation-based inference steps.**

## Major points:

**1) To be fair, one should compare the results of the MMD test to those obtained through another nonparametric multivariate (or univariate, respectively) 2-sample test, whereas the Hotelling T-squared (or t-test, respectively) is parametric and requires normality and homoskedasticity.**

The original authors of the MMD method did an extensive comparison of their test with other multivariate two-sample statistical tests, both on synthetic and real data. They showed that the test we use (MMD^2 B) is the best in most cases (some of the results can be seen on table below, from the paper entitled **A Kernel Two-Sample Test** by Greton A, et al, https://jmlr.org/papers/volume13/gretton12a/gretton12a.pdf, section 8). We added a mention of this result in the last supplementary section.

| Data Set | Attr. | $MMD_b$ | $MMD_u^2$ H | $MMD_u^2$ B | $MMD_u^2$ M | t-test | Wolf | Smir | Hall |
|---|---|---|---|---|---|---|---|---|---|
| Neural Data I | Same | 100.0 | 100.0 | 96.5 | 96.5 | 100.0 | 97.0 | 95.0 | 96.0 |
| | Different | 38.0 | 100.0 | **0.0** | **0.0** | 42.0 | **0.0** | 10.0 | 49.0 |
| Neural Data II | Same | 100.0 | 100.0 | 94.6 | 95.2 | 100.0 | 95.0 | 94.5 | 96.0 |
| | Different | 99.7 | 100.0 | 3.3 | 3.4 | 100.0 | **0.8** | 31.8 | 5.9 |
| Health status | Same | 100.0 | 100.0 | 95.5 | 94.4 | 100.0 | 94.7 | 96.1 | 95.6 |
| | Different | 100.0 | 100.0 | 1.0 | **0.8** | 100.0 | 2.8 | 44.0 | 35.7 |
| Subtype | Same | 100.0 | 100.0 | 99.1 | 96.4 | 100.0 | 94.6 | 97.3 | 96.5 |
| | Different | 100.0 | 100.0 | **0.0** | **0.0** | 100.0 | **0.0** | 28.4 | 0.2 |

Table 1: Distribution testing for data integration on multivariate data. Numbers indicate the percentage of repetitions for which the null hypothesis (p=q) was accepted, given $\alpha = 0.05$. Sample size (dimension; repetitions of experiment): Neural I 4000 (63; 100) ; Neural II 1000 (100; 1200); Health Status 25 (12,600; 1000); Subtype 25 (2,118; 1000).

| Data Set | Attr. | $MMD_b$ | $MMD_u^2$ H | $MMD_u^2$ B | $MMD_u^2$ M | t-test | Wolf | Smir | Hall | Biau |
|---|---|---|---|---|---|---|---|---|---|---|
| BIO | Same | 100.0 | 100.0 | 93.8 | 94.8 | 95.2 | 90.3 | 95.8 | 95.3 | 99.3 |
| | Different | 20.0 | 52.6 | **17.2** | 17.6 | 36.2 | **17.2** | 18.6 | 17.9 | 42.1 |
| FOREST | Same | 100.0 | 100.0 | 96.4 | 96.0 | 97.4 | 94.6 | 99.8 | 95.5 | 100.0 |
| | Different | 3.9 | 11.0 | **0.0** | **0.0** | 0.2 | 3.8 | **0.0** | 50.1 | **0.0** |
| CNUM | Same | 100.0 | 100.0 | 94.5 | 93.8 | 94.0 | 98.4 | 97.5 | 91.2 | 98.5 |
| | Different | 14.9 | 52.7 | 2.7 | **2.5** | 19.17 | 22.5 | 11.6 | 79.1 | 50.5 |
| FOREST10D | Same | 100.0 | 100.0 | 94.0 | 94.0 | 100.0 | 93.5 | 96.5 | 97.0 | 100.0 |
| | Different | 86.6 | 100.0 | **0.0** | **0.0** | **0.0** | **0.0** | 1.0 | 72.0 | 100.0 |

Table 2: Naive attribute matching on univariate (BIO, FOREST, CNUM) and multivariate (FOREST10D) data. Numbers indicate the percentage of times the null hypothesis $p = q$ was accepted with $\alpha = 0.05$, pooled over attributes. Sample size (dimension; attributes; repetitions of experiment): BIO 377 (1; 6; 100); FOREST 538 (1; 10; 100); CNUM 386 (1; 13; 100); FOREST10D 1000 (10; 2; 100).

**2) The experiments described in the article involve the comparison of more than two conditions. In this case, a 2-sample test can be used as a post-hoc pairwise analysis (with the proper correction for multiple comparisons) after carrying out a (significant) nonparametric omnibus test, whereas the authors apply directly the MMD test pairwise. Please discuss this point and propose a procedure for these cases.**

Omnibus tests are usually conducted to target the post-hoc 2-sample tests realized downstream and limit their number (which will be used later on when adjusting for the number of performed comparisons e.g. with a Bonferroni correction). Here (as in most cases where we resorted to MMD-based statistical tests), the p-values are so low that we do not need such a strategy to limit the number of comparisons. In cases where one suspects that the p-values will be close to the significance threshold (small effect, low number of trajectories), we advise to carefully pick the performed 2-sample comparisons by selecting only the relevant pairs of conditions/groups. We added a sentence about this at the end of the subsection introducing the statistical test.

**3) I can agree with the authors that different architectures showing similar performance in an inference or classification task learn an equivalent latent representation. However, I believe that the relevant question here is how the choice of the learning bias influences the latent representation. For example, I would expect the method to behave differently if one would use different (or additional, or less) target features for training with respect to model probability and alpha.**

In our case, the latent space is always structured, irrespective of whether we train the network to predict alpha, the RW type or both simultaneously. This property is not a predefined property of graph neural networks (GNNs) but a consequence of the specific questions addressed in this paper. We did numerical simulations where we show the

latent representations of trajectories obtained with networks trained on both tasks or on just one of them. Colors in the top row correspond to the type of random walk, and in the bottom row to the anomalous diffusion exponent (blue: alpha < 1, red: alpha > 1). The structure is clearly influenced by the objective (see for instance below how the space of a network trained to infer alpha is almost 1-dimensional). We chose to train the model on both the classification and the regression of the anomalous exponent because both these factors are relevant criteria and the network should learn to discriminate trajectories according to both. We show here a total of 10^5 trajectories of length 15.

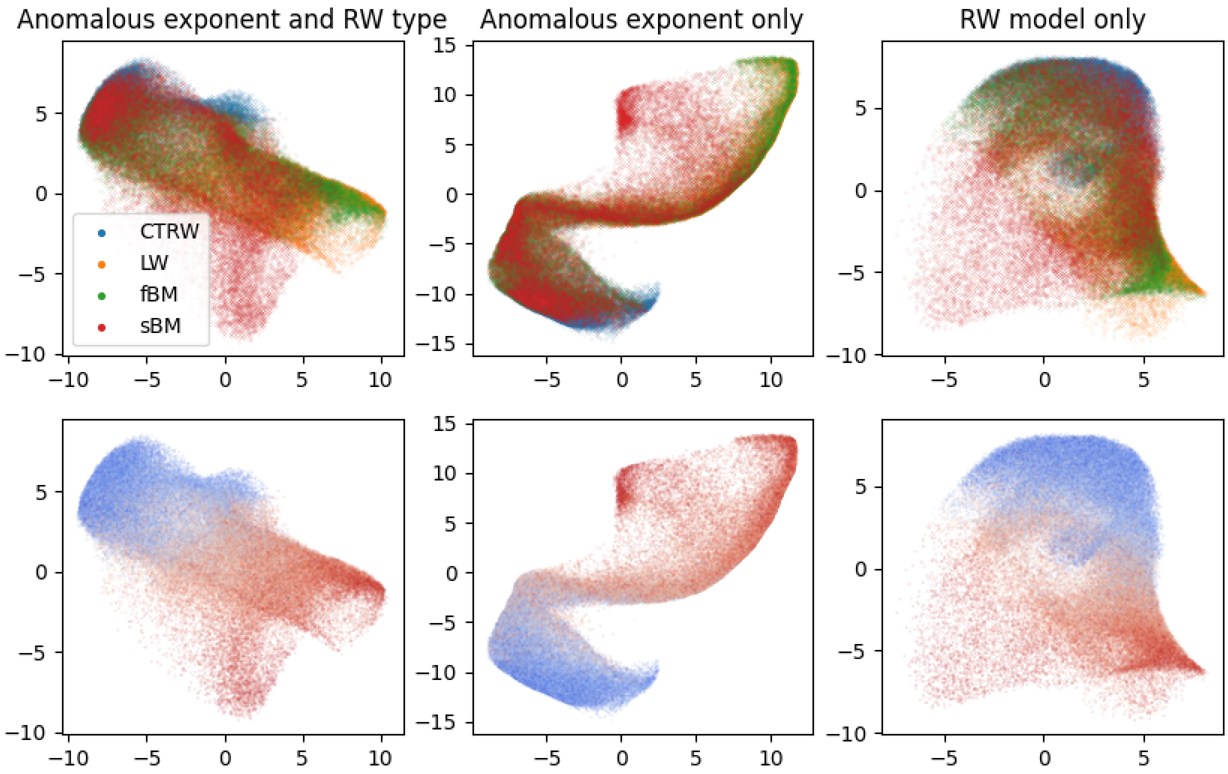


**Or if one would replace the supervised GNN with an architecture with no additional target features besides trajectory coordinates and time. What would the machine learn in these cases? How would the latent representation change? Just to give an example, one might think to obtain the latent representation by using autoencoders (as in https://iopscience.iop.org/article/10.1088/1751-8121/ac3786) or even a generative model (e.g., variational autoencoders).**

We agree there is a form of no free lunch theorem (see https://arxiv.org/abs/2202.04513 for example): other training methods might also be able to make the network learn meaningful representations of trajectories. Nevertheless, auto-encoders are not optimal for our purposes, as they would tend to either learn “superfluous” information, not strictly related to properties of the generative model, or would require huge amounts of training data to extract information from the noise-dominated SMT data. A very challenging practical problem is that the point-to-point euclidean distance between two trajectories can be very large even if they were both generated by the same random walk model, forcing the latent representation to incorporate information about the precise path taken by the diffusing particle, while we want it to focus as much as possible on the diffusion process itself. A potential solution could be to train the model to predict future steps of the trajectory, maybe with a variational framework (the network would thus learn to infer the propagator from the trajectory). However, the point still remains that while it might be possible to train an unsupervised latent embedding, the supervised objectives provide efficient inductive biases.

**4) I wonder how much the choice of the dataset used for the training contributes to implicitly defining the latent features that are extracted. I guess it would be interesting to investigate the influence of the parameter**

**space used in the training dataset. I believe that some comparison (e.g., using different training sets and the same test set) might help to partly clarify this point.**

We performed numerical simulations in our first paper about GNNs for trajectory analysis (JPA Verdier 2021, Fig. 10 F, https://iopscience.iop.org/article/10.1088/1751-8121/abfa45/meta) where we provided Ornstein-Uhlenbeck trajectories as input to the network, while this model of motion was absent from the training sets. We observed that they were projected close to the borders of the fBM with alpha < 1 in the latent space, a physically reasonable position as this motion is anticorrelated due to the confinement effect of the harmonic potential. Here, we performed another subset of simulations where we show latent representation of a same set of trajectories obtained with networks trained with/:

- Only fBM,
- Only sBM,
- CTRW and LW.

We show results below, where each of the four types of random walk has a distinctive latent distribution: for instance, the model trained only on sBM produces a different distribution for LW than it does for fBM and would thus be capable, using the method we present in the article, to distinguish between those.

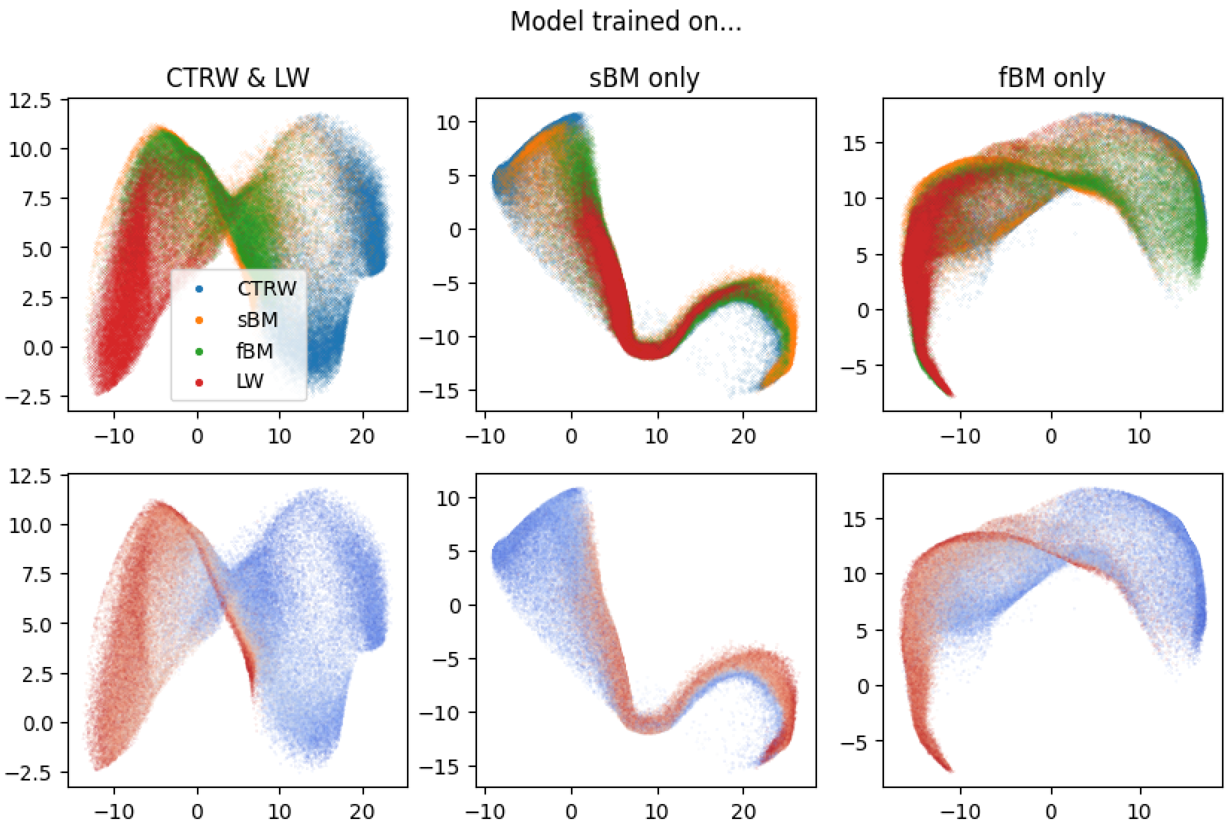


We included this figure and the one used to answer your point #3 as a Supplementary figure 5 in the revised manuscript.

**5) Are the learned features correlated? The results shown in fig S4B (Gratin2 vs Gratin16) seem to suggest so. Please discuss the possible implications for the statistical test.**

Yes, some features are correlated. But it has no impact on the significance of the statistical test since we do not measure a difference between dimensions, but between sets of points, regardless of the manifold they lie on.

**6) Are the learned features correlated with the analytical indicators (before UMAP reduction)? This might provide hints about the interpretability of the features.**

Yes, they are indeed. Here is a correlation matrix. It furthermore confirms that there is some redundancy in the latent space features.

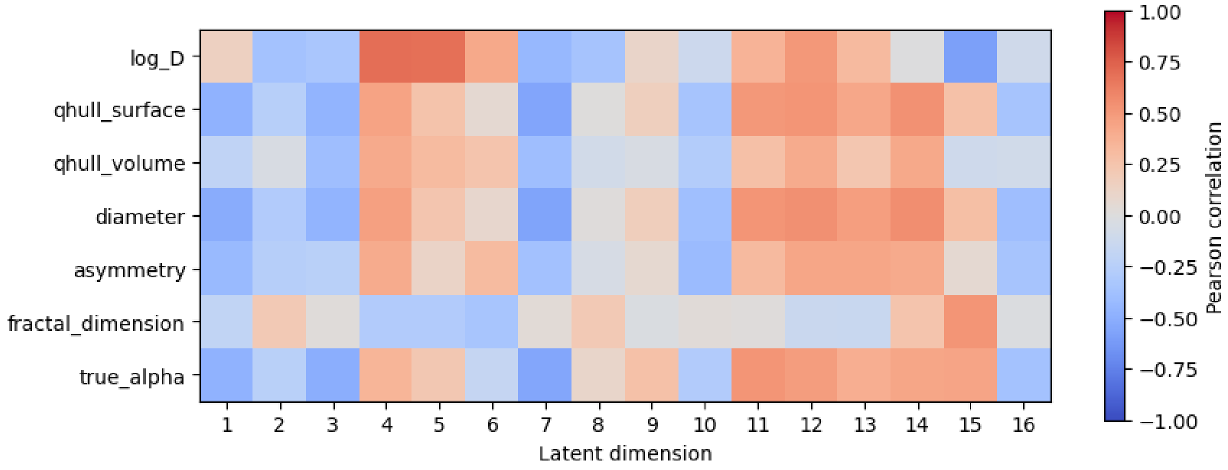


# Minor points:

**1) In the reply, The authors stated “We also added a reference to the method of Muñoz-Gil et al. 2021, which, like ours, relies on learnt representations of individual trajectories” but I could not find it in the references of the revised manuscript.**

Thanks for pointing this out. We intended to add a sentence in the introductory part referencing this study. We have now included one.

**2) There is a missing reference on line 816**

Fixed, thanks.

**3) I find it confusing to use \alpha both for the anomalous diffusion exponent and the significance level of the statistical test**

Indeed. We changed to using \beta for the significance level of the test.

# Reviewer #2

***(In black, your comments,*** *in purple, our answers* ***)***

**The authors did a good job in revising their manuscript, but a few points in their response are rather obscure. In point 1 they claim that "Very often, observed biomolecules do not all exhibit the same type of dynamics ... " which I must interpret as a statistical ensemble of paths (and a probability measure on said space of paths) does not exists. Few sentences later they claim that their method can "identify the most closely corresponding random walk model" which directly (and irrefutably) contradicts the previous statement. I know that the probabilistic "hygiene" is not the main priority, but the authors should try being a bit more coherent in their claims.**

We were unclear indeed. In the first quote, we meant “observed biomolecules *within a set of experimentally observed ones”,* that is, a set of trajectories can be a mixture of several types of RW. The supervised method identifies, per individual trajectory, the closest RW model (not per set of trajectories).

**Moreover, stationary is not an assumption that one must make, one can test for that (directly, and rather straightforwardly). Scientists have been (quantitatively) describing observations of glassy behavior long before the advent of machine learning.**

Indeed, but observations of glassy behavior are usually longer than the trajectories we consider here by many orders of magnitude.

**I do agree that the "dealing with under-sampled data" may be a strength of the method and this could potentially be made even more visible.**

**Something I did not mention in my original report (but the authors may consider) is to classify trajectories of the "unequal twin" processes using a small number of trajectories (https://journals.aps.org/prl/abstract/10.1103/PhysRevLett.107.260601). This may be a challenge but would (potentially) also objectively demonstrate the power of the new method. But this is optional.**

Fractional Brownian motion and scaled Brownian motion have the same marginal PDF and our method is able to distinguish them well even with low numbers of trajectories (see Fig S.2). Thank you for pointing this out. We added a sentence in the results section and a reference to the work you mention to emphasize this interest.
